# Characteristics of idiopathic pulmonary fibrosis -associated cough. a case-control study

**DOI:** 10.1186/s12890-023-02476-7

**Published:** 2023-05-23

**Authors:** Eeva Saari, Minna Mononen, Hannele Hasala, Anne Lätti, Johanna Kaulamo, Hanna Nurmi, Riitta Kaarteenaho, Minna Purokivi, Heikki O Koskela

**Affiliations:** 1 Division of Respiratory Medicine, Institute of Clinical Medicine, School of Medicine, Faculty of Health Sciences, University of Eastern Finland, 1627, 70211 Kuopio, PO Finland; 2 Center of Medicine and Clinical Research, Division of Respiratory Medicine, Kuopio University Hospital, PO 100, 70029 Kuopio, Finland; 3 Department of Respiratory Medicine, Tampere University Hospital, PO 2000, 33521 Tampere, Finland; 4 Health Care Services for Prisoners, Mehiläinen Terveyspalvelut Oy, Kauppakatu 39A, 70110 Kuopio, Finland; 5 Research Unit of Internal Medicine, University of Oulu, PO 500, 90400 Oulu, Finland; 6 Center of Internal Medicine and Respiratory Medicine and Medical Research Center (MRC) Oulu, Oulu University Hospital, PO 20, 90029 Oulu, Finland

**Keywords:** Idiopathic pulmonary fibrosis (IPF), Cough, Leicester Cough Questionnaire (LCQ)

## Abstract

**Background:**

Most patients with idiopathic pulmonary fibrosis (IPF) complain of cough. IPF-associated cough is widely characterized as dry or non-productive. The aim of this study was to compare chronic cough in early stage IPF patients to cough in subjects with chronic cough from a community-based sample and, especially, to investigate whether cough in IPF is less productive than chronic cough in a community-based sample.

**Methods:**

The IPF cough population consisted of 46 biopsy-confirmed patients who complained of chronic cough. Control population consisted of subjects with chronic cough, gathered by a community-based email survey sent to public service employees and the Finnish Pensioners’ Federation. A case-control setting was applied by having four age, gender, and smoking-status matched subjects from the community sample for each IPF cough patient. A cough specific quality of life questionnaire (Leicester Cough Questionnaire (LCQ)) was filled in by all subjects. The LCQ questionnaire contains 19 questions, each question is scored from 1 to 7 and total score from 3 to 21 with a smaller value indicating more severe impairment.

**Results:**

The sputum production frequency, as assessed by LCQ question 2, was 5.0 (3.0-6.0) in the IPF chronic cough population and 5.0 (3.0–6.0) in the community-based chronic cough population (median and interquartile range *p*= 0.72). The LCQ total score was 14.8 (11.5-18.1) in the IPF chronic cough population and 15.4 (13.0–17.5) in the community-based chronic cough population (*p*=0.76). The domain impact scores were physical, 4.9 (3.9–6.1) *vs*. 5.1 (4.5–5.6) (*p*=0.80); psychological, 4.6 (3.7–5.9) *vs*. 4.7 (3.9–5.7) (*p*=0.90); and social, 5.5 (3.7–6.5) *vs*. 5.5 (4.5–6.3) (*p*=0.84), respectively. Furthermore, cough response to paint or fumes, cough disturbing sleep, and cough frequency per day did not differ between the groups.

**Conclusion:**

Cough in early stage IPF patients was not distinguishable from chronic cough in the community-based population by LCQ. Especially, there was no difference in the self-reported frequency of cough-associated sputum production.

## Background

Cough is the most common symptom for seeking medical advice, worldwide [1]. Successful management of chronic cough implies identification of the background disorder maintaining the cough and offering an efficient therapy against it [2]. Idiopathic pulmonary fibrosis (IPF) is one, though rare, possible background disorder for cough. IPF is a chronic, progressive, fibrotic interstitial lung disease (ILD) with an unknown cause. IPF prognosis after diagnosis is only 3-5 years depending on the variable course of the disease [3]. Due to the progressive disease course of IPF, it is useful to recognize early signs of the disease. Early diagnosis is needed in order to start anti-fibrotic medication and to slow down disease progression. The first symptoms of IPF include exertional dyspnea and cough [4]. The prevalence of cough among IPF patients varies between 75-85 % [4–7]. Cough is associated with impairment of quality of life (QoL) and it also predicts the time to hospitalization, death and lung transplantation [8]. IPF cough is commonly characterized as dry or non-productive [9–12]. However, to the best of our knowledge, there are no previous controlled studies about this issue.

The primary aim of this study is to investigate whether cough in early stage IPF is less productive than chronic cough in a community-based population. We also investigated whether there are differences between these populations with respect to the Leicester Cough Questionnaire (LCQ) total score, domain scores, and four special questions raised from the LCQ: cough response to paint or fumes, cough disturbing sleep, cough frequency per day, and sputum production.

## Methods

### Setting

This study consisted of two populations: an IPF chronic cough (IPF cc) population and a community-based chronic cough (community-based cc) population. Case-control setting was applied and for each IPF patient with chronic cough, four age-, gender- and smoking-status matched controls were selected from the community-based population with chronic cough. Chronic cough was defined as a cough that has lasted more than eight weeks [13].

### IPF chronic cough population

**Fig. 1:**
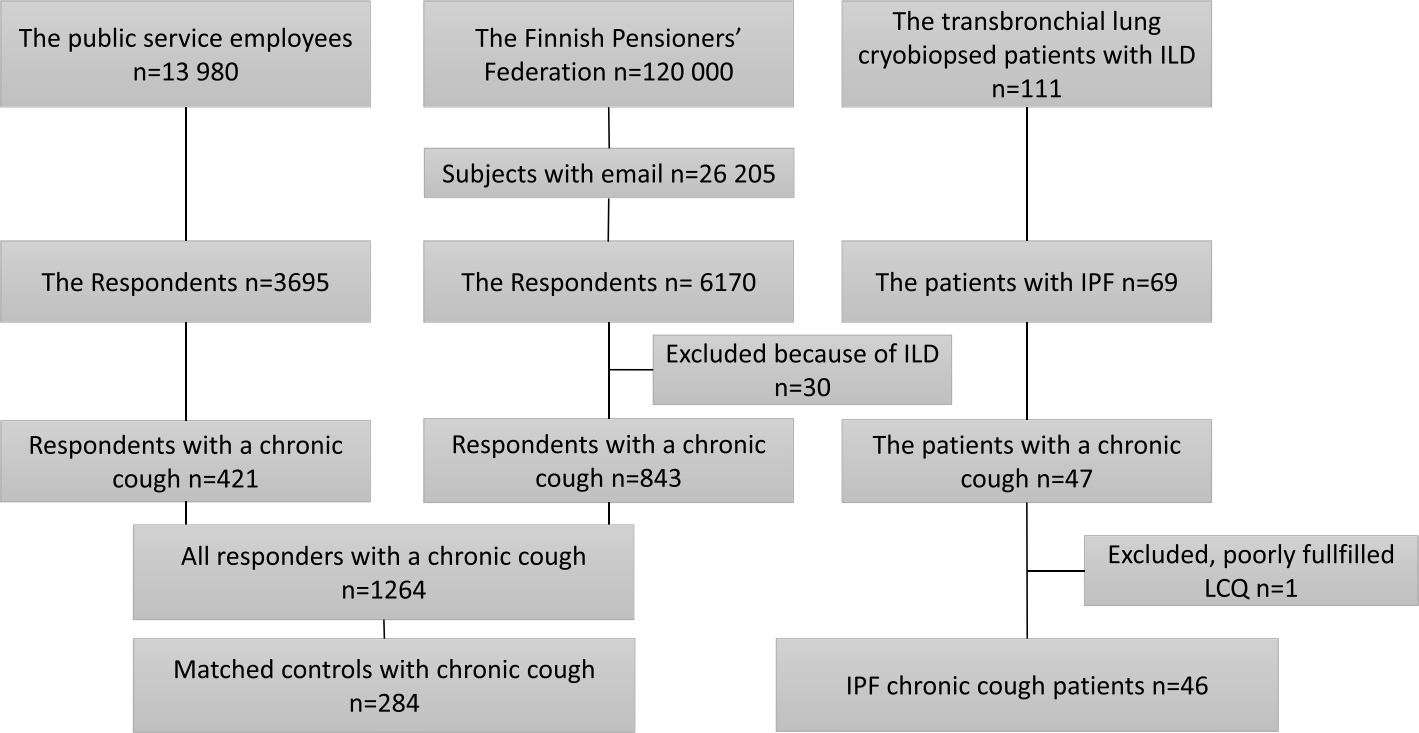
Flowchart of the study populations

The patients with IPF were prospectively recruited from Kuopio University Hospital (KUH) and Tampere University Hospital (TAUH) pulmonology clinics between January 2015 and December 2021 (Fig. 1). There were 111 patients with suspected ILD. All participants underwent HRCT-, and a transbronchial lung cryobiopsy (TBLC) was needed to confirm the diagnosis. The exclusion criteria included forced vital expiratory volume in 1 s (FEV1) < 50%, total lung capacity < 50%, diffusion capacity to carbon monoxide (DLCO) < 50%, mean pulmonary artery pressure >55 mmHg in echocardiogram (ECHO), body mass index (BMI) > 30 kg/m^2^ if the mean pulmonary artery pressure in ECHO was > 55 mmHg. The rest of the exclusion criteria are described in more detail in an earlier publication [14]. Multidisciplinary discussions (MDD) were held before the specific diagnosis on ILD were set, as recommended by the international guidelines at the time [15, 16]. Forty-seven out of 69 (68 %) IPF patients complained of chronic cough at the first visit. One patient was excluded due to an insufficiently filled in LCQ (Fig. 1). Patient data with medication, comorbidities, and lung functions (forced vital capacity (FVC), DLCO) were collected from the electronic medical records. The LCQ was collected at the baseline before TBLC. Age was calculated at the time when the LCQ was filled out. Gender-Age-Physiology (GAP) index was calculated using gender, age and two lung physiology variables (FVC and DLCO) [17]. A written, informed consent was obtained from all participants. This prospective study protocol was approved by the Research Ethics Committee of the Northern Savo Hospital district (statement 80/2014) and the Tampere University Hospital (R15149). This study was conducted in compliance with the Declaration of Helsinki. The collection of the data is described in detail in an earlier publication [14].

### Community-based chronic cough population

The control subjects with chronic cough had participated in either of the two, large community-based surveys conducted in 2017 and 2021. The first survey was conducted on public service employees in the cities of Kuopio and Jyväskylä, including 13 980 employees (Fig. 1). The second survey was conducted on the Finnish Pensioners’ Federation, including 26 205 members, with available email addresses (Fig. 1). Both surveys were conducted via email and recorded in electronic datasheets which included the LCQ. A reminder email was sent 2 weeks later if there had been no response. A response to the questionnaire was considered as an informed consent. Both studies were approved by the Research Ethics Committee of the Northern Savo Hospital district (statement 289/2015). The studies are described in more detail in earlier publications [2, 18].

### LCQ and GAP index

LCQ is a well validated, cough specific quality of life (QoL) questionnaire. It contains 19 questions; of which each is scored from 1 to 7 with one indicating the most severe impairment. The following four LCQ questions were analyzed in detail: sputum production, cough response to paints or fumes, cough disturbing sleep, and cough bout frequency per day. The total LCQ score ranges from 3 to 21, representing the sum of the three different domains: physical, psychological, and social impacts of cough [19].

GAP index predicts one-, two- and three-year mortalities in IPF. GAP index points 0-3 present the lowest mortality stage I with one year mortality 5.6 %, points 4-5 present stage II with one year mortality 16.2 %, and points 6-8 present stage III with one year mortality 39.2 %[17].

### Statistical analysis

For each IPF patients with chronic cough, four age-, gender-, and smoking status-matched controls were selected from the subjects reporting chronic cough in either of the two community-based surveys. Age was matched ±4 years. Smoking status was matched in consideration of ever smoking. Patients with fibrotic lung disease were excluded from the community-based population to avoid overlap. There were 46 IPF cases with chronic cough and 184 matched controls with chronic cough. The statistical power of analysis was 86% for the detection of 0.5 SD-unit difference between the groups with an alpha-significance level of 0.05.

The LCQ total score, three different domain scores and the four special questions raised from the LCQ were used to specify characteristics of IPF chronic cough from community-based chronic cough. The distributions of these variables differed significantly from the normal distributions (one-sample Kolmogorov-Smirnov Test). Therefore, a non-parametric Mann-Whitney U test was utilized to compare the IPF patients and the controls. Categorical variables were compared by Chi-square or Fisher's exact tests.

The data is presented as frequencies with percentages or medians and interquartile ranges. P < 0.05 was accepted as a level for statistical significance. All data was analyzed using IBM SPSS Statistics version 26 and R statistical software version 4.04 for the statistical analyses.

## Results

The prevalence of chronic cough was 68 % among the subjects with IPF. The comparison of the 46 IPF cc patients and the 22 IPF patients who did not complain of cough is presented in Table 1. The former group had a significantly lower cough related QoL measured by the LCQ. Also, the scores of cough disturbing sleep and cough bout frequency per day were significantly lower among the IPF population complaining of cough. The female IPF patients reported cough significantly more often than the male patients (Table 1). However, the cough specific QoL did not significantly differ between genders (LCQ total score 14.8 (11.3–18.7) for females and 17.8 (14.2–19.3) for males, *p*=0.773) among the total IPF population. GAP index was calculated and 83% of the IPF population belonged to GAP stage I and only eight patients belonged to GAP stage II.

**Table 1:** Comparison between the IPF patients complaining of cough to those who did not.

Measured variable	Total IPF-population (*n*=68)	IPF cc population (*n*=46)	IPF-population without cough (*n*=22)	*p*-value
Age	67.0 (62.3–72.8)	67.5 (64.7–70.0)	67 (61.0–75.0)	0.870
Gender (female)	29 (42.6 %)	26 (56.5%)	3 (13.6 %)	0.001
BMI (kg/m2)	28.1 (26.4–31.5)	27.9 (26.7–30.8)	28.5 (26.4–30.6)	0.911
Current smoker	6 (8.8 %)	5 (10.9 %)	1 (4.5 %)	0.390
Ever smoker	42 (61.8 %)	28 (60.9%)	14 (63.6 %)	0.826
FVC (% predicted)	79.5 (72.3–88.0)	78.50 (73.7–82.3)	84.5 (77.0–91.25)	0.098
DLCO (% predicted)	64.0 (53.5–73.0)	63.50 (58.3–68.0)	64.0 (46.6–73.5)	0.637
GAP Index	3.00 (2.00–3.00)	3.00 (2.00–3.00)	3.00 (2.00–4.00)	0.352
Sputum production	5.0 (3.0–6.0)	5.0 (3.0–6.0)	6.0 (4.00–7.00)	0.075
Cough response to paint or fumes	6.0 (5.0–7.0)	5.5 (5.0–7.0)	6.0 (6.00–7.00)	0.057
Cough disturbing sleep	6.0 (5.0–7.0)	5.5 (4.0–6.0)	7.0 (6.00–7.00)	0.000
Cough bout frequency per day	5.0 (3.0–6.0)	4.0 (3.0–6.0)	6.0 (5.75–7.00)	0.000
LCQ total score	16.9 (13.4–18.9)	14.8 (11.5–18.1)	18.2 (16.4–19.4)	0.002
LCQ physical domain	5.4 (4.5–6.1)	4.9 (3.9–6.1)	5.9 (5.1–6.4)	0.003
LCQ psychological domain	5.2 (4.1–6.3)	4.6 (3.7–5.9)	6.0 (5.0–6.7)	0.002
LCQ social domain	5.8 (4.8–6.7)	5.5 (3.7–6.5)	6.4 (5.8–6.8)	0.006

Values are given as medians with interquartile ranges, *p*-value is represented between the IPF-population with and without cough

*IPF* Idiopathic pulmonary fibrosis, *cc* Chronic cough, *BMI* Body mass index, *FVC* Forced vital capacity, *DLCO* Diffusion capacity for carbon monoxide, *GAP* Gender Age Physiology Index, *LCQ* Leicester Cough Questionnaire

The main characteristics of the IPF cc and community-based cc populations are presented in Table 2. The groups were matched with respect to age, gender, and smoking status. Also, the BMI was similar in both populations. There were no missing values in the data.

**Table 2:** Baseline characteristics of the IPF cc population and the community-based cc population

Characteristic	IPF cc population (*n*=46)	Community-based cc population (*n*=184)
Age (years)	67.5 (64.7–70.0)	67.5 (64.0–70.0)
Gender (female)	26 (56.5 %)	104 (56.5 %)
BMI (kg/m2)	27.9 (26.7–30.8)	27.4 (25.9–29.4)
Current smoker	5 (10.9 %)	13 (7.1 %)
Ever smoker	28 (60.9 %)	112 (60.9 %)
FVC (% predicted)	78.50 (73.7–82.3)	N/A
DLCO (% predicted)	63.50 (58.3–68.0)	N/A
GERD	3 (6.5 %)	N/A
OSA	2 (4.3 %)	N/A
Asthma	6 (13.0 %)	N/A
COPD	0	N/A

Values are given as medians with interquartile ranges or absolute numbers n with percentages in parenthesis.

*IPF* Idiopathic pulmonary fibrosis, *cc* Chronic cough, *BMI* Body mass index, *FVC* Forced vital capacity, *DLCO* Diffusion capacity for carbon monoxide, *GERD* Gastro esophageal reflux disease, *OSA* Obstructive sleep apnea, *COPD* Chronic obstructive pulmonary disease, *N/A* Not applicable

**Fig. 2:**
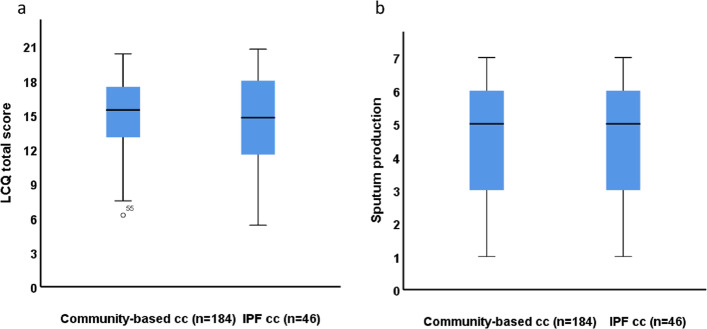
LCQ total score (**a**) and sputum production score (**b**) between community-based cc and IPF cc Values are shown as medians with interquartile ranges.

The main findings are presented in Table 3. The frequency of sputum production during coughing was similar in both populations (Fig. 2). Furthermore, there were no significant differences between the IPF cc and community-based cc in cough response to paint or fumes, cough disturbing sleep or cough bout frequency per day. Also, the LCQ total scores (Fig. 2) and the LCQ physical, psychological, and social impact scores did not differ significantly between the populations.

**Table 3:** Comparison of the IPF cc population and the community-based cc population

Measured variable	IPF cc population (*n*=46)	Community-based cc population (*n*=184)	*p*-value
Sputum production	5.0 (3.0–6.0)	5.0 (3.0–6.0)	0.72
Cough response to paint or fumes	5.5 (5.0–7.0)	6.0 (4.0–7.0)	0.77
Cough disturbing sleep	5.5 (4.0–6.0)	6.0 (4.0–6.0)	0.87
Cough bout frequency per day	4.0 (3.0–6.0)	5.0 (4.0–5.0)	0.20
LCQ total score	14.8 (11.5–18.1)	15.4 (13.0–17.5)	0.76
LCQ physical domain	4.9 (3.9–6.1)	5.1 (4.5–5.6)	0.80
LCQ psychological domain	4.6 (3.7–5.9)	4.7 (3.9–5.7)	0.90
LCQ social domain	5.5 (3.7–6.5)	5.5 (4.5–6.3)	0.84

Values are given as medians with interquartile ranges

*IPF* Idiopathic pulmonary fibrosis, *LCQ* Leicester Cough Questionnaire, *cc* Chronic cough

There was a total of ten (*n*=10) patients in the IPF cc population with common comorbidities causing cough (asthma *n*=6, gastro esophageal reflux disease (GERD) *n*=3, obstructive sleep apnea (OSA) *n*=2, Table 2). There were no significant differences in the LCQ scores or special questions when comparing IPF cc patients with comorbidities to the rest of the IPF cc population. However, the IPF cc patients with comorbidities had lower LCQ physical domain scores (median 4.32 (3.69–5.10) *vs.* 5.26 (4.19–6.13) *p*=0.066).

## Discussion

To the best of our knowledge, this is the first controlled study about the characteristics of IPF-associated cough. A case-control setting was utilized to minimize the effects of age, gender, and smoking status and to increase the statistical power of the study. The results suggested that in early stage IPF disease cough is undistinguishable from a community-based chronic cough. Furthermore, there were no differences in the cough related QoL between the IPF cc and the community-based cc groups compared by the LCQ total score, and its three domains: the physical, psychological, and social impact scores. Moreover, the specific LCQ questions regarding sputum production, cough response to paint or fumes, cough disturbing sleep, and cough bout per day revealed no differences between the groups. Our study showed that female IPF patients are more prone to report cough than male IPF patients, but the difference is no longer evident when measured by the LCQ.

The frequency of cough-associated sputum production was identical in both chronic cough groups as measured by LCQ. This finding is notable since IPF cough is often considered as dry or non-productive by an expert opinion or general assumption [4, 10, 11, 20]. However, this conception is not based on controlled studies. In the present study, the sputum production of IPF cc patients was for the first time compared with that of a matched community-based sample of subjects with chronic cough. Our study does not support the widely hypothesized dry or non-productive cough in IPF.

The LCQ total score or domain scores did not differ between the two populations in our study. The IPF cc population’s LCQ total score and domain scores were quite similar to the study of Key et al., showing a median LCQ total score of 15.4 and median domain cough impact scores of 5.13 for physical, 5.29 for psychological, and 5.75 for social in 19 IPF patients [20]. Scholand et al studied 68 IPF patients with mucin 5B (MUC5B) polymorphism revealing a LCQ total score of 16.16 ± 3.66, physical 5.24, psychological 5.46, and social 5.46 [21]. Our LCQ scores were roughly equal to Key et al but more severe than those of Scholand et al. These differences are most probably due to patient selection, since we included IPF patients who had complained of cough and excluded those without cough, while Key et al included IPF patients with typical IPF findings in spirometry and HRCT, without focusing on the presence or absence of cough. Scholand and co-authors also included patients irrespective of symptomatic cough. Lee et al conducted a large ILD study with 1,447 participants 61.3% of them were IPF patients. They measured a mean LCQ total score of 16.5 ± 3.7 with no disease-specific differences (IPF, non-IPF, connective tissue disease-associated (CTD) ILD, and hypersensitivity pneumonitis (HP)). They showed a lower impact to the cough related QoL in ILD than our study but did not report the domain scores [8]. A Japanese study on ILD cohort analyzed cough among a population of idiopathic interstitial pneumonia (IIP) (*n*=70) including IPF 51.4 %, HP and CTD-ILD. They did not find any significant differences in LCQ scores, although cough severity and frequency were worse in the IIP group using the cough visual analog scale [22].

History of cough sensitivity to paints and fumes is well known to associate with objectively measured cough reflex hypersensitivity [23–25]. Therefore, this question of the LCQ was analyzed in detail in the present study. Our results showed that sensitivity to paints and fumes does not differ between early stage IPF cc patients and population-based cc patients, suggesting a similar degree of cough reflex sensitivity between these groups.

The early stage IPF chronic cough did not seem to disturb sleep more often than the chronic cough in the community-based sample. Two previous studies have suggested that IPF cough is more prevalent during the day, with relatively little nocturnal cough [20, 26]. However, the above-mentioned studies had no control population and they included only 19 and 9 IPF patients, respectively.

Cough bout frequency per day was similar in both groups in this study. Key et al. have shown earlier that IPF patients have a higher cough frequency than healthy subjects and asthmatic subjects but a similar cough frequency to those with chronic cough presenting to a specialist clinic [20]. Our study showed that cough frequency per day was similar in the early stage IPF cc and the community-based cc population and is in line with the data of Key et al. with a wider community-based control population [20].

In the present study, female IPF patients complained of chronic cough significantly more often than males. However, the cough related QoL did not differ significantly between the genders. This discrepancy might be due to females’ tendency to express their cough verbally more often compared to males. A previous study has also suggested that female IPF patients report cough more often than male patients, and has shown that presence of cough or phlegm was negatively associated with transplant-free survival in men but not in women [27]. Considering the latter result, asking carefully about coughing in male IPF patients at an appointment seems necessary. 

Ten IPF patients in our study had other comorbidities associated with cough (asthma, GERD and OSA) which seemed to have a trend to affect the LCQ physical impact score. However, it did not quite reach the statistical significance in this small IPF population. Previous studies have shown that GERD, OSA and asthma are common in the IPF population and they affect the QoL of IPF patients [28–30]. This finding is in line with that of our study. One quarter of our IPF cough population had comorbidities while three quarters did not.

The first limitation of our study was the mild severity of IPF patients. It has been shown that cough in IPF is associated with advanced disease [5]. There might have been differences in the results if IPF patients with more advanced disease were included. However, the study setting corresponds the situation of the real life in which patients seek medical attention due to recently appeared symptoms. Secondly, our IPF patients with cough were not the most typical IPF patients due to inclusion and exclusion criteria of the study. Patients were included by the need of TBLC which excluded IPF patients with typical HRCT findings. In addition, IPF patients were excluded, if they were not suitable for the TBLC procedure due to poor lung function or BMI >30. Finally, there is a possibility of the type 2 statistical error secondary to too small populations.

The controlled setting can be regarded as a strength of the present study. It provides a good possibility to investigate whether a general physician can recognize IPF patients by the nature of their cough from the community-based cough at a doctor’s appointment. Also, the diagnostics of patients were solid since all IPF patients were biopsy-proven, and the MDD were held as suggested by the current guidelines at the time before the IPF diagnosis was set. Furthermore, the control group was large and well matched by age, gender and smoking status, giving sufficient power to this study to examine statistical differences in the study population. The LCQ is a well validated cough quality of life questionnaire and all the study patients had filled in the questionnaires completely.

Since chronic cough in the IPF population was not distinguishable from the community-based chronic cough, it is essential to examine cough patients carefully during primary care visits to promptly find serious background diseases, like IPF. Lung auscultation, chest x-ray and critical thinking after a failed cough treatment should be done for all patients complaining of chronic cough. The possibility of IPF should be remembered also in patients with productive cough.

## Conclusion

Cough in early stage IPF patients was not distinguishable from chronic cough in the community-based population by LCQ. Especially, there was no difference in the self-reported frequency of cough-associated sputum production. According to this study, the widely accepted concept of a non-productive cough in IPF was shown to be inaccurate. Also, other characteristics of cough in the IPF population were similar to those in the community-based population. Further studies containing objective measures of cough severity and with a wider range of the disease severity and more typical HRCT findings are needed to better characterize IPF associated cough.

## Data Availability

The datasets used and analyzed during the current study are available from the corresponding author on reasonable request.

## Abbreviations

BMI: body mass index
cc: chronic cough
CTD: connective tissue disease
DLCO: diffusion capacity to carbon monoxide
ECHO: echocardiogram
FVC: forced vital capacity
GAP: gender age physiology
GERD: gastro esophageal reflux disease
HRCT: high-resolution computed tomography
ILD: interstitial lung disease
IPF: idiopathic pulmonary fibrosis
KUH: Kuopio university hospital
LCQ: Leicester cough questionnaire
MDD: multidisciplinary discussion
OSA: obstructive sleep apnea
TAUH: Tampere university hospital
TBLC: transbronchial lung cryobiopsy
